# Prescription opioids induced microbial dysbiosis worsens severity of chronic pancreatitis and drives pain hypersensitivity

**DOI:** 10.1080/19490976.2024.2310291

**Published:** 2024-02-08

**Authors:** Kousik Kesh, Junyi Tao, Nillu Ghosh, Richa Jalodia, Salma Singh, Rajinder Dawra, Sabita Roy

**Affiliations:** Department of Surgery, Sylvester Comprehensive Cancer Center, University of Miami, Miami, FL, USA

**Keywords:** Chronic pancreatitis, gastrointestinal microbiome, opioid, morphine, oxycodone, pain

## Abstract

Opioids, such as morphine and oxycodone, are widely used for pain management associated with chronic pancreatitis (CP); however, their impact on the progression and pain sensitivity of CP has never been evaluated. This report investigates the impact of opioid use on the severity of CP, pain sensitivity, and the gut microbiome. C57BL/6 mice were divided into control, CP, CP with morphine/oxycodone, and either morphine or oxycodone alone groups. CP was induced by administration of caerulein (50ug/kg/h, i.p. hourly x7, twice a week for 10 weeks). The mouse-to-pancreas weight ratio, histology, and Sirius red staining were performed to measure CP severity. Tail flick and paw pressure assays were used to measure thermal and mechanical pain. DNA was extracted from the fecal samples and subjected to whole-genome shotgun sequencing. Germ-free mice were used to validate the role of gut microbiome in sensitizing acute pancreatic inflammation. Opioid treatment exacerbates CP by increasing pancreatic necrosis, fibrosis, and immune-cell infiltration. Opioid-treated CP mice exhibited enhanced pain hypersensitivity and showed distinct clustering of the gut microbiome compared to untreated CP mice, with severely compromised gut barrier integrity. Fecal microbiota transplantation (FMT) from opioid-treated CP mice into germ-free mice resulted in pancreatic inflammation in response to a suboptimal caerulein dose. Together, these analyses revealed that opioids worsen the severity of CP and induce significant alterations in pain sensitivity and the gut microbiome in a caerulein CP mouse model. Microbial dysbiosis plays an important role in sensitizing the host to pancreatic inflammation.

## Introduction

Chronic pancreatitis (CP) is characterized by progressive destruction of the pancreatic secretory parenchyma and its replacement by fibrous tissue, leading to impaired exocrine and endocrine functions.^1^ Known causes of CP include long-term exposure to alcohol, smoking, metabolic and vascular diseases, and autoimmune or genetic disorders. However, in a significant proportion of CP cases, labeled sporadic or idiopathic, the underlying cause remains unclear.^1–3^ The annual incidence of CP ranges from 5 to 14 per 100,000 individuals worldwide, with a prevalence of approximately 30–50 per 100,000 individuals.^4^ Functional consequences of CP include recurrent or constant abdominal pain, pancreatic exocrine insufficiency, and diabetes.^1,2^ Pain is present in most patients, and pain-associated disability distinctly reduces their quality of life and ultimately leads to early death. CP is associated with primary pancreatic pain (duct obstruction, tissue hypertension, active inflammation, and altered nociception), secondary pain (local and remote complications), and treatment-related pain from surgical procedures or medications.^4^

Treatment follows the principles of the ‘pain relief ladder’ provided by the World Health Organization (WHO),^5^ with the serial introduction of drugs with increasing analgesic potency from non-opioid analgesics such as nonsteroidal anti-inflammatory drugs, weak opioids, and potent opioids. However, in patients with severe and debilitating pain patterns, simple analgesics are insufficient, and a more aggressive approach using opioids combined with adjuvant analgesics as first-line therapy is useful to control pain and prevent sensitization of the central pain pathway.^6^ While opioids are used to manage CP pain, opioid treatment has its own adverse effects. In fact, according to a retrospective study of 176,857 CP patients, opioid use disorder in CP patients had doubled from 2.7% in 2005 to 5.4% in 2014.^7^ Chronic opioid use, even when used appropriately, causes hyperalgesia and induces bowel dysfunction in patients.^8,9^ Opioid use has also been known to result in gut dysbiosis in various human and preclinical models.^10,11^ There are reports the alteration in the gut microbiome in CP,^12–15^ but in these studies, the gut microbiome in CP progression in the context of opioids has not been evaluated. In the current study, we characterized opioid-associated alterations in the gut microbiome during experimental CP, as it relates to CP severity and pain sensitivity, using a caerulein CP mouse model.

## Results

### The establishment of caerulein-induced chronic pancreatitis (CP) model

**Figure 1.**
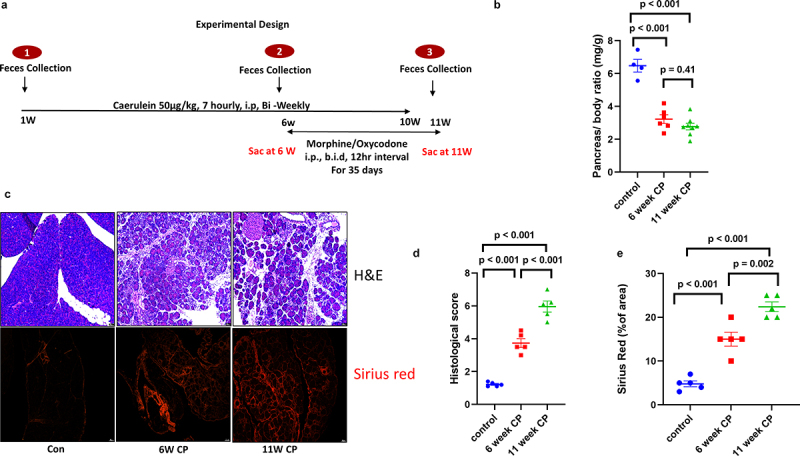
Progression of CP in a caerulein-induced mouse model: (a) schematic of caerulein administration for developing a mouse model of CP and treatment with opioids to understand their effect on CP. (b) Pancreas to mouse weight ratio at 6^th^ and 11^th^ week as compared to control group. (c) Representative H &E and Sirius red stain of control, 6W CP and 11W CP mice. Pancreases revealed increased atrophy and collagen deposition with increased duration of caerulein treatment. Histological representation of pancreas atrophy (d) and percent of Sirius red stained area in mice pancreas. (e) Data were analyzed by t-tests (two-tail). ***p < .001. Data are represented as mean ± SD.

An experimental CP model was induced in C57BL/6 male mice by repeated intraperitoneal injections of caerulein for 10 weeks. Figure 1(a) shows the schematic of the experiment. The progression of CP was monitored by euthanizing a group of caerulein-treated mice at 6 weeks. Pancreatic atrophy, calculated as the pancreas-to-mouse weight ratio, and pancreatic fibrosis were measured to validate the model. Pancreas to mouse weight ratio was significantly (*p* < .05) reduced in the 6-week CP groups compared to the control, indicating CP development. Pancreas to body weight ratio at week 10 was significantly lower than that at week 0 (*p* < .05) and tended to be lower than that at week 6 (*p* =.06) (Figure 1(b)). Histological analysis revealed steady loss of acinar cells during CP progression. The infiltration of immune cells also increased with CP progression. Pancreatic fibrosis, a key marker of CP, also increased significantly during disease progression (Figure 1(c–e)).

### Gut microbiome change during the development of the model

**Figure 2.**
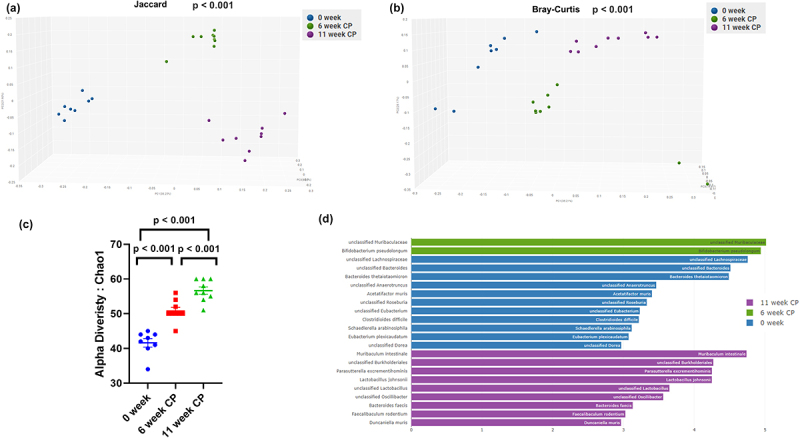
Diversity and composition analysis of the fecal microbiome samples. Samples are grouped by 0-week control (*n* = 9), 6-week CP (*n* = 9) and 11-week CP (*n* = 9). (a) Principal coordinates analysis (PCoA) plot of Jaccard distance (metrics of β -diversity). P-value <.001 between any two groups. (b) PCoA plot of Bray-Curtis distance (metrics of β -diversity). P-value < .001 between any two groups. (c) Chao11 index (metrics of α-diversity). Error bars represent SEM. P-value = .034 between 0-week and 6-week CP. P-value = .001 between 0-week and 11-week CP. P-value = .003 between 6-week and 11-week CP. (d) LefSeSe (linear discriminant analysis effect size) analysis of top discriminative bacteria species between samples from 0-week control, 6-week CP, and 11-week CP. LDA threshold > 3.0.

In the caerulein CP mouse model, altered microbiome diversity and composition were observed as early as 6 weeks. When β diversity was measured using both Jaccard and Bray-Curtis distances and visualized with principal-coordinate analysis (PCoA) plots, fecal samples from 0-week control mice significantly clustered apart from mice at 6-week or 10-week CP (*p* < .01) (Figure 2(a,b)). Fecal samples from mice at 6-week CP were also significantly different from mice on 10-week CP (*p* < .01) (Figure 2(a,b)). The α-diversity measured by CHAO1 was lowest in fecal samples from healthy controls and highest in fecal samples from mice with 11-week CP (*p* < .001) (Figure 2C). α-diversity was also significantly lower in fecal samples from mice with 6-week CP than in fecal samples from mice with 11-week CP (*p* < .001) (Figure 2(c)). Higher α-diversity demonstrated that more unique bacterial taxa were present in the gut of mice that developed CP. Together, these analyses revealed pronounced changes in the gut microbial diversity when mice developed CP. LefSe (Linear Discriminant Analysis (LDA) Effect Size) analysis was also performed among samples to determine the bacterial taxa that were differentially enriched. Indeed, the plot of the LDA score also confirmed that compared to the 0-week control group, *Muribaculum intestinale* and *Lactobacillus Johnsonii* were more enriched in the 11-week CP samples (Figure 2(d)). *Bifidobacterium pseudolongum* was abundant in the 6-week CP sample compared to the 0-week and 11-week samples (Figure 2(d)).

### Opioid treatment increased pancreatic atrophy and fibrosis in a caerulein mice model of CP

**Figure 3.**
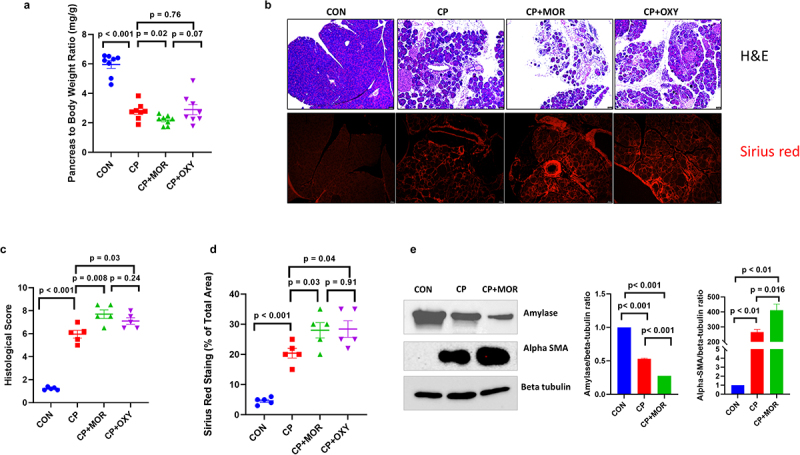
Opioid treatment after induction of CP causes increased pancreatic atrophy, inflammation, and fibrosis: (a) pancreas to mouse weight ratio shows a significant reduction in CP and CP + opioid groups compared to control. CP + MOR groups showed further significant reduction of pancreas to mouse weight ratio compared to CP group. (b) Representative H&E and Sirius red stain of control, 11W CP, 11W CP + MOR, and 11W CP + OXY mice pancreases revealed increased atrophy and collagen deposition in CP and opioid-treated CP mice group. Histologic score of pancreatic atrophy in different groups (c) and percent of Sirius red stained area in mice pancreas (d). Data were analyzed by t-tests (two-tail). ***p< .001. Data are represented as mean ± SD, with n = 8 in each group. (e) Western blots showed the expression of a-Amylase, a-SMA and beta- tubulin in control, 11W CP, 11W CP+MOR, pancreatic tissue.

The effect of opioids on pancreatic injury and fibrosis was evaluated in a caerulein CP mouse model. Mice in the opioid group were injected (*i.p*) with escalating doses of either morphine (10, 20, 30, 40, 50 mg/kg) or oxycodone (5, 15, 25, 35, 45 mg/kg) twice a day during the last 5 weeks of caerulein injection. H&E and Sirius red staining of pancreatic tissues were performed to evaluate the inflammatory and fibrotic effects (Figure 3). CP at 11 weeks without opioid treatment showed significant morphological damage in the form of increased inflammatory infiltrate, fibrosis, and a reduced pancreas-to-mouse weight ratio. As illustrated in Figure 3(a), the relative pancreas weight of the CP group was significantly lower than that of the control group (*p* < .05). In the CP + MOR group, a further decrease in the relative pancreas weight was observed (*p* < .05), indicating that morphine potentiates pancreas atrophy induced by repetitive injections of caerulein. Similarly, the pancreatic pathological score and positive area of Sirius red staining were also markedly increased in the opioid-treated group compared to the saline-treated CP group (Figure 3(b-d)). Expression of a-Amylase, an indicator of acinar cell mass, showed a significant decrease in CP groups compared to control. Further, amylase level was even lower in with opioid treatment in CP+morphine group (Figure 3(e)). The pancreatic stellate cells activation markers a-SMA were significantly increased with CP treatment compared to control (Figure 3(e)). Moreover, a-SMA was significantly higher with morphine treatment in CP+morphine compared to CP group (Figure 3(e)). Morphine or oxycodone treatment alone did not show any significant changes in pathological score compared to control except for some edema formation (Figure S1).

### Opioid treatment increases intestinal damage during CP

Morphine and CP are known to compromise gut barrier function independently.^16,17^ Since intestinal permeability with bacterial translocation has been demonstrated to cause systemic injury during CP, we evaluated whether a combination of CP and opioid treatment would further augment gut barrier disruption. We found that the CP combined with opioid treatment groups showed increased gut barrier disruption (Figure S2). The severity of damage was much higher in opioid-treated CP mice than in the CP-only groups (*p* < .05) (Figure S2C). We also investigated the tight junction organization of intestinal epithelium by staining for tight junction proteins Claudin-1. In control mice, Claudin-1 staining is continuous and localized to the apical side of the intestinal epithelial membrane (Figure S10). In contrast, CP treatment led to disrupted Claudin-1 organization, suggesting impaired recruitment of tight junction proteins to the membrane (Figure S10). Additionally, more disruption of Claudin-1 was observed in mice with CP+morphine treatment, when compared to the CP and control (Figure S10).

### Morphine treatment increases CP-associated hyperalgesia pain

**Figure 4.**
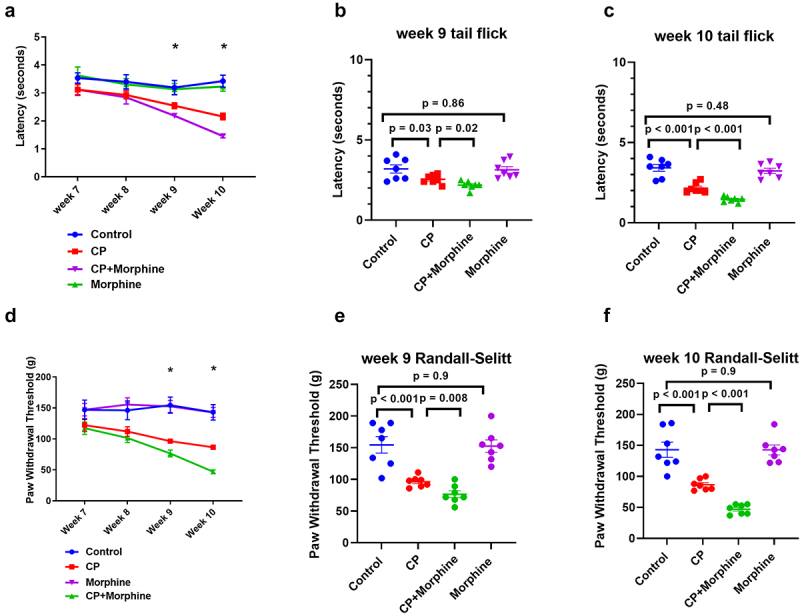
Morphine increased thermal and mechanical pain sensitivities in caerulein-induced CP mice: (a) thermal pain sensitivity was measured by tail flick test from week 7 to week 10. (b) Thermal pain sensitivity at week 9. (c) Thermal pain sensitivity at week 10. (d) Mechanical pain sensitivity was measured by Randall-Selitto test from week 7 to week 10. (e) Mechanical pain sensitivity at week 9. (f) Mechanical pain sensitivity at week 10.

Thermal and mechanical pain sensitivity in all groups were measured at the end of weeks 7, 8, 9, and 10 before morphine injection (Figure 4). CP-only groups had increased pain sensitivity to thermal stimuli compared to the control (Figure 4(a–c)) (*p* < .05). Moreover, the morphine-treated CP groups demonstrated significantly greater sensitivity to thermal stimuli, with decreased withdrawal latencies (Figure 4(a–c)) (*p* < .05) at 9 and 10 weeks compared to CP. Similarly, the CP-only groups had greater pain sensitivity to mechanical stimuli than the control (Figure 4(d–f)) (*p* < .05). CP mice injected with morphine also demonstrated increased sensitivity to mechanical stimuli, with decreased withdrawal latencies (Figure 4(d–f)) (*p* < .05). There was no significant change in the withdrawal latencies observed in the control mice compared to the morphine-only treatment group (*p* > .05).

### Opioid treatment further alters microbiome composition and function during CP

**Figure 5.**
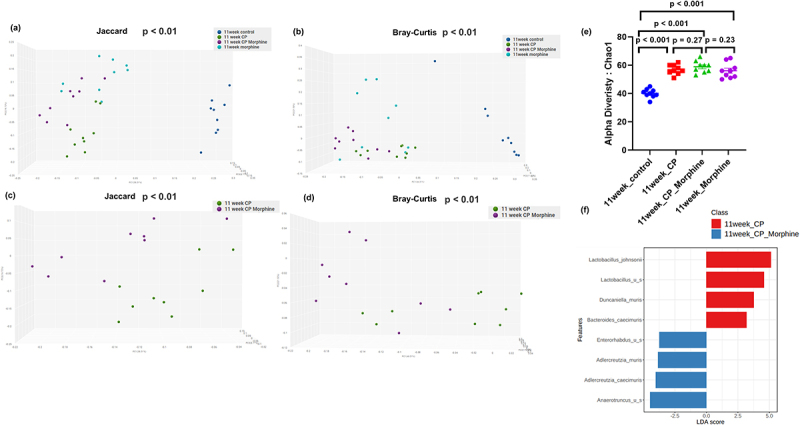
Diversity and composition analysis of fecal microbiome samples. Samples are grouped by 11-week control (*n* = 9), 11-week CP (*n* = 9), 11-week CP + morphine (*n* = 9), and 11-week morphine (*n* = 9). (a) PCoA plot of Jaccard distance (metrics of β -diversity) in all groups. P-value < .01 between any two groups. (b) PCoA plot of Bray-Curtis distance (metrics of β -diversity) in all groups. P-value < .01 between any two groups. (c) PCoA plot of Jaccard distance (metrics of β -diversity) in 11-week CP and 11-week CP + morphine. P-value < .01 between 11-week CP and 11-week CP + morphine. (d) PCoA plot of Bray-Curtis distance (metrics of β -diversity) in 1- week CP and 11-week CP + morphine. P-value < .01 between 11-week CP and 11-week CP + morphine. (e) Chao11 index (metrics of α-diversity). Error bars represent SEM. P-value < .001 between 11-week control and 11-week CP, 11-week CP + morphine, or 11-week morphine. P-value > .05 between 11-week CP, 11-week CP + morphine, and 11-week morphine. (f) LefSeSe (linear discriminant analysis effect size) analysis of top discriminative bacteria species between 11-week CP and 11-week CP + morphine. LDA threshold > 2. “u s” is short for “unidentified species”.

Altered microbiome diversity, composition, and function in morphine-treated CP animals were observed in 11-week samples. When β diversity was measured using both Jaccard and Bray-Curtis distances and visualized with PCoA plots, fecal samples from control mice significantly clustered distinctly from the CP mice, morphine, or CP + morphine treatment groups (*p* < .01) (Figure 5(a,b)). Moreover, fecal samples from CP + morphine mice were also significantly different from those from CP-only mice at 11 weeks (*p* < .01) (Figure 5(c,d)). Similar β-diversity results were observed in oxycodone-treated CP animals in 11-week samples (Figure S3(a–d)).

The α-diversity measured by CHAO1 was lower in fecal samples from healthy controls than in those from CP, Morphine, and CP + morphine mice with 11-week CP (*p* < .001) (Figure 5(e)). The α-diversity was not significantly different in fecal samples between mice treated with CP, Morphine, and CP + morphine (*p* > .05) (Figure 5(e)). Higher α-diversity demonstrated that more unique bacterial taxa were present in the gut of mice that developed CP or were treated with morphine. Similar α-diversity results were observed in oxycodome-treated CP animals in 11-week samples (Figure S3(e)). A heatmap of microbial taxa at the species level in the 11-week CP and 11-week CP + morphine samples was plotted to highlight the effect of morphine on CP (Figure S4(a)). LefSe (Linear Discriminant Analysis (LDA) Effect Size) analysis was also performed between the CP and CP + morphine samples to determine the bacterial taxa that were differentially enriched. The bar plot of the LDA score demonstrated that unidentified species from *Anaerotruncus*, *Adlercreutzia caecimuris*, and *Adlercreutzia muris* were enriched in 11-week CP morphine compared to CP-only (Figure 5(f)). *Lactobacillus johnsonii*, an unidentified species from *Lactobacillus*, and *Ducaniella muris* were enriched in 11-week CP (Figure 5(f)). A heatmap of microbial taxa at the species level in 11-week CP and 11-week CP + oxycodone samples was plotted to highlight the effect of oxycodone on CP (Figure S4(b)).

LefSe analysis was also performed between the CP and CP + oxycodone samples to determine the bacterial taxa that were differentially enriched. LefSe analysis identified similar but not identical species that were differentially enriched in the 11-week CP + oxycodone group. *Adlercreutzia muris*, unidentified species from *Enterorhabdus*, and *Adlercreutzia mucosicola* were enriched in the 11-week CP + oxycodone group. Unidentified species from *Muribaclaceae*, *Bacteroides faecis*, and *Ducaniella muris* were enriched in 11-week oxycodone (Figure S3(f)).

**Figure 6.**
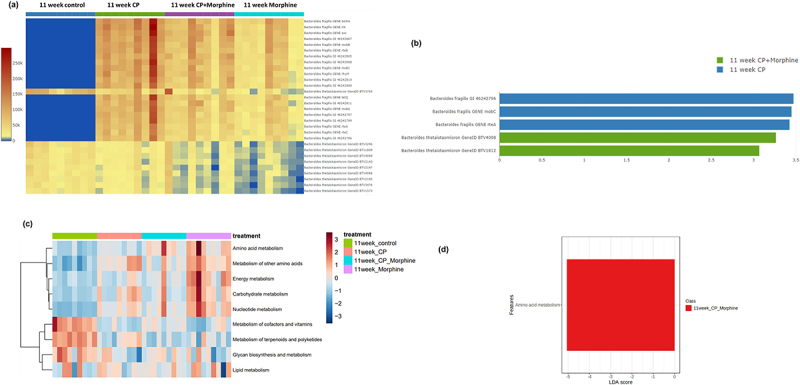
Function pathway analysis of the fecal microbiome samples. Samples are grouped by 11-week control (*n* = 9), 11-week CP (*n* = 9), 11-week CP + morphine (*n* = 9), and 11-week morphine (*n* = 9). (a) Heatmap of virulence factors in all groups. (b) LefSeSe (linear discriminant analysis effect size) analysis of top discriminative virulence factors between 11-week CP and 11-week CP + morphine. LDA threshold > 2. (c) Heatmap of KEGG pathways in all groups. (d) LefSeSe (linear discriminant analysis effect size) analysis of top discriminative KEGG pathways between 11-week CP and 11-week CP + morphine. LDA threshold > 2.

Virulence-associated genes were also matched using the Cosmos ID database, and the results are summarized in Figure 6 and Table S2. Control samples lacked almost all *Bacteroides fragilis*-related virulence factors but had a higher abundance of *Bacteroides thetaiotaomicron-*related virulence factors. LefSe analysis demonstrated that *Bacteroides fragilis* GI 46,343,796, mobC, and rteA were more abundant in the 11-week CP group than in the 11-week CP + Morphine group (Figure 6(b)). *Bacteroides thetaiotaomicron* BTV 4098 and BTV 1812 were more abundant in the 11-week CP + morphine group (Figure 6(b)).

Similar results were observed for the oxycodone samples with virulence-associated genes (Figure S5). Control samples were absent of all *Bacteroides fragilis-*related virulence factors, but had a higher abundance of *Bacteroides thetaiotaomicron*-related virulence factors. LefSe analysis demonstrated that Bacteroides fragilis GI 46,343,796 and rteA were more abundant in 11-week CP (Figure b. Virulence factors were not more abundant in the 11-week CP + oxycodone group (Figure S5(b)).

The MetaCyc pathway, GO terms, and KEGG pathways are summarized in Supplemental Table S4-S6. A KEGG pathway heatmap of the morphine samples is shown in (Figure 6(c)). From the KEGG pathways, general amino acid metabolism was elevated in 11-week CP Morphine compared to that in 11-week CP (Figure 6(d)). Similarly, MetaCyc pathways showed that the super pathway of branched amino acid biosynthesis, L-isoleucine biosynthesis, and L-lysine biosynthesis were upregulated in 11-week CP + morphine (Figure S6(a)). Queuosine biosynthesis, urea biosynthesis/inosine 5’ phosphate degradation, and guanosine ribonucleotides de novo biosynthesis were upregulated in 11-week CP (Figure S6(a)). The GO terms ATP binding, recombinase activity, and phosphorelay signal transduction system were more abundant in the 11-week CP + morphine group (Figure S6(b)). The translation elongation factor activity and RNA-directed DNA polymerase activity were more abundant in the 11-week CP group (Figure S6(b)).

A KEGG pathway heatmap of the oxycodone samples is shown in (Figure S5(c)). Additional KEGG pathways were identified to be differentially expressed. General amino acid metabolism, metabolism of cofactors and vitamins, glycan biosynthesis and metabolism, and lipid metabolism were elevated in the 11-week CP + oxycodone group over the 11-week CP group (Figure S5(d)). Carbohydrate and nucleotide metabolism were more active in 11-week CP than in 11-week CP + oxycodone. Similar MetaCyc pathways and GO terms were identified in oxycodone samples (Figure S7(a,b)).

### Morphine and oxycodone altered the microbiome similarly during CP

To investigate whether morphine and oxycodone have similar effects on the microbiome, we compared the diversity between morphine and oxycodone using control samples and CP samples as a baseline.

Both morphine and oxycodone induced alteration in microbiome diversity of control samples. When β-diversity was measured using Jaccard distances and visualized with PCoA plots, morphine-treated fecal samples were significantly different from oxycodone-treated samples (*p* = .028) (Figure S8(a)). However, when β diversity was measured using Bray-Curtis distances, fecal samples from morphine-treated mice were no longer significantly different from those in oxycodone-treated mice (*p* = .23) (Figure S8(b)). The α-diversity measured by CHAO1 was lower in fecal samples from the control than morphine or oxycodone with 11-week CP (*p* < .001) (Figure S8(c)). However, α-diversity was not significantly different in the fecal samples between morphine and oxycodone (*p* = .72) (Figure S8(c)). LEfSe analysis identified *Faecalibacterium rodentium* to be significantly higher in oxycodone than in morphine group (Figure S8(d)).

Similarly, both morphine and oxycodone altered microbiome diversity in CP samples. When β-diversity was measured using both Jaccard and Bray-Curtis distances and visualized with PCoA plots, fecal samples from morphine-treated mice were not significantly different from those of oxycodone-treated mice (*p* = 0.23) (Figure S9(a,b)). The α-diversity measured by CHAO1 was not significantly different between the CP, CP + morphine, or CP + oxycodone groups (*p* > .05) (Figure S9(c)). LEfSe analysis did not identify any significantly different feature between CP + morphine and CP + oxycodone.

In summary, morphine and oxycodone had similar effects on the microbiome, especially in the context of CP.

### The morphine-induced microbiome sensitized caerulein-induced acute pancreatic inflammation in germ-free mice

**Figure 7.**
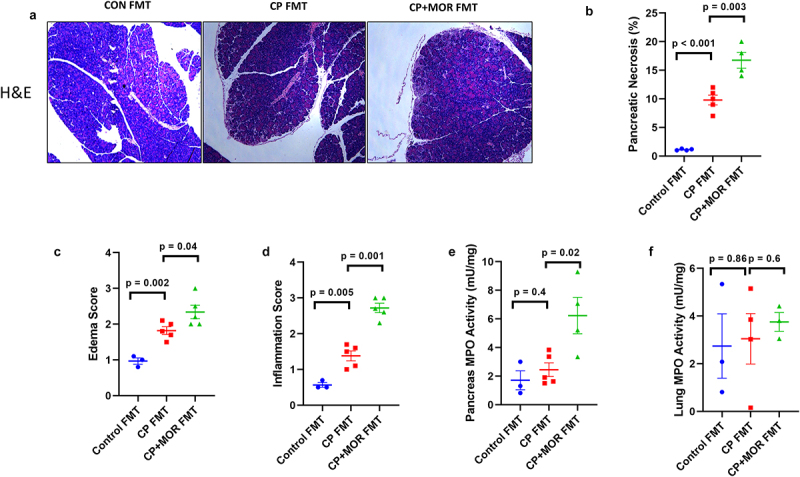
The morphine-induced microbiome sensitized caerulein-induced acute pancreatic inflammation. Germ-free mice were gavaged with control, CP, and CP + MOR mice microbiome followed by suboptimal caerulein challenge. (a) Representative H&E staining of mice pancreas histology of different groups, demonstrating that CP and CP + morphine microbiota sensitize to pancreatic injury at suboptimal caerulein dose (5ug/kg/h) as indicated by increased inflammation and necrosis, when compared with control microbiota gavage mice. Quantification of the ratio of pancreatic necrosis, (b) edema, (c) and infiltrating immune cells (d). MPO activity of pancreases (e and lung extract (f) of caerulein-treated microbiome gavage of germ-free mice. Data were analysed by t tests (two-tail). ***p < .001. Data are represented as mean ± SD, with *n* = 5 in each group.

To investigate whether administering morphine-induced gut microbiota to germ-free mice would sensitize the mice to sub-optimal dose of caerulein (5ug/kg/h)-induced pancreatic injury and inflammation, we first established a fecal microbiota transplantation (FMT) in germ-free mice. FMT from control, CP, and morphine-treated CP mice was performed in germ-free mice. Germ-free mice that received FMT from CP or CP + MOR showed a significant increase in pancreatic injury as measured by necrosis, edema, and inflammation using a suboptimal dose (5 μg/kg/h x7) of caerulein (Figure 7(a–d)). Germ-free mice that received control mice FMT did not show any pancreatic inflammation upon a suboptimal level of caerulein (5ug/kg) treatment (Figure 7(a–d)), similar to wild-type mice (data not shown). Moreover, germ-free mice that received CP + MOR FMT displayed more damage than those received CP FMT (Figure 7(a–d)), indicating the deleterious effect of morphine-induced dysbiotic microbes in increasing pancreatic susceptibility to injury. Furthermore, CP + MOR FMT also significantly increased pancreases’ myeloperoxidase (MPO) activity compared with CP or control FMT (*p* < .05) (Figure 7(e)). There was no significant difference in lung MPO activity (*p* < .05) (Figure 7(f)), although there was a numeric increase in MPO activity.

## Discussion

Chronic pancreatitis (CP) contributes significantly to morbidity and mortality, resulting in a substantial financial burden on society and decreased quality of life for individuals. CP is a fibro-inflammatory disease characterized by various local and systemic complications, including chronic pain. Patients with CP may be asymptomatic for long periods. As the disease progresses, patients may experience unrelenting and severe abdominal pain, requiring hospitalization. Opioid analgesics are commonly used to treat severe CP-associated pain; however, the effect of opioids on the severity of CP has not been investigated. A previous study by our group delineated the underlying mechanisms and side effects of opioids in an acute pancreatitis model.^18^ In the current study, we investigated the effects of two opioids (morphine and oxycodone) on the severity of CP and the underlying mechanisms of opioid-associated pancreatic inflammation. Herein, we report that opioid treatment results in an increase in the severity of CP and the associated pain. This causes gut dysbiosis, which may aggravate pancreatic inflammation. As CP is a painful disease that frequently requires higher doses of narcotic analgesics, the choice of opioids for analgesia in CP has been a topic of much debate. It is unclear whether opioid treatment, when compared to non-opioid analgesics, leads to worsening of the disease. Moreover, there are no data linking these physiological mechanisms with the clinical outcome of worsening pain or disease severity. In the caerulein CP mouse model, we found that pancreatic atrophy and fibrosis (the commonly used parameters of CP) were significantly different at 6-week after caerulein treatment. The disease was further established with continued caerulein administration for up to 10 weeks, as revealed by the evaluation of pancreatic changes in animals at the 11th week. Morphine and oxycodone aggravated the disease, as evidenced by increased atrophy, fibrosis, and decreased pancreas weight and animal weight ratio in opioid-treated groups compared to caerulein-alone treatment (Figures 1 and 3). We previously reported that morphine worsens severity and prevents pancreatic regeneration in mouse models of acute pancreatitis (AP).^18^ Similarly, Bálint et al. also reported that morphine decreased vacuolization in edematous AP, while buprenorphine pretreatment increased pancreatic edema.^19^ Here, for the first time, we report that morphine and oxycodone increase the severity of CP in a preclinical setting. The current study raises an important concern regarding whether prolonged opioid administration drives hyperalgesia or allodynia in patients with CP. Previous studies have documented that administration of high opioid doses can evoke an allodynic/hyperalgesia state in preexisting disease conditions.^20^ Paradoxically, opioid therapy aimed at alleviating CP pain may cause increased sensitivity to pain and potentially aggravate preexisting pain.

The gut microbiome is increasingly recognized for its role in pancreatic disease.^21–23^ A recent study suggested that CP is associated with dysbiosis either directly or indirectly.^12^ The potential roles and mechanisms of action of gut microbiota in CP remain to be fully elucidated. Our results are consistent with most clinical^3,13,24,25^ and preclinical studies,^12,14^ and we observed significant changes in α and β-diversity when mice developed CP, in parallel with the significant microbiome change in CP patients.^3,13,24,25^ Moreover, we have also shown that alterations in microbiome diversity and composition can be observed as early as 6 weeks (Figure 2). One study also observed changes in the mouse microbiome diversity at 6 weeks. However, a different model with an ethanol and caerulein combination was used in that study.^14^ It is well established that both acute and chronic exposure to alcohol can extensively modify microbiome composition and function; therefore, using alcohol in a mouse study is in itself a confounding factor.^26^ We demonstrated that early alteration of the microbiome was independent of ethanol treatment. We observed higher α-diversity with the development of CP, which is consistent with the results of our previous study.^12^ However, our results differed from those of previous studies in the field. In human patients with CP, a decrease in α-diversity was reported when compared to healthy individuals.^3,13,24,27^ A decrease in α-diversity has also been reported in patients with chronic alcoholic pancreatitis.^28^ One clinical study reported no difference in richness or diversity between patients with CP and healthy controls; however, pancreatic samples were used instead of fecal samples.^29^ Another clinical study demonstrated that patients with diabetes secondary to CP (Type 3c) had similar α-diversity (Chao1 index and Fischer’s alpha) as control subjects, but higher than those with type 1 and 2 diabetes.^25^ One study using a combination of ethanol and caerulein to induce CP showed decreased bacterial richness and diversity in a mouse model.^14^ The contrasting findings in α-diversity could be due to a different model for inducing CP or alcohol and tobacco consumption in clinical studies. Finally, we identified bacterial taxa that were differentially enriched in CP to the species level using a shotgun metagenomics approach, whereas previous studies used only variable regions (V1-V2, V4, and V5-V6) of the 16S rRNA gene. Sequencing with variable regions has only a reliable resolution at the genus level. Species-level resolution is more informative for future efforts to modify the microbiome. We have identified that *Muribaculum intestinale*, *Lactobacillus johnsonii*, and *Bifidobacterium pseudolongum* were abundant in the CP samples. *Muribaculum intestinale* is a strictly anaerobic bacterium which has been isolated from the cecal content of a mouse, as part of the mouse intestinal bacterial collection. It is capable of degrading galactose, pectin, glucosamine, and other metabolites.^30^ *M. intestinale*’s ability to utilize a variety of nutrients likely gives it survival advantage in the progression of CP, when the availability of nutrients increased with decreased digestive enzyme secretion. In another report, *M. intestinale* was reduced prior to the onset of ileitis in a genetically modified mouse model, indicating it may play a role in other types of inflammation disease.^31^ *Lactobacillus johnsonii strain 456* and *LA1* are used as probiotics for reducing *Helicobacter pylori* colonization in human and reducing systemic inflammation in mice,^32,33^ but strain LA1 was ineffective in preventing endoscopic recurrence of Crohn’s disease in a clinical study.^34^ *Lactobacillus johnsonii* has also been reported to produce hydrogen peroxide, which can kill other competitive bacteria in the vicinity.^35^ The ability to produce hydrogen peroxide could be one of the reasons they are more abundant in CP samples, however, whether the production of hydrogen peroxide contributes to the progression of the CP remains to be investigated. Bo et al., found that *B. Pseudolongum* supplementation has fat-reducing effect on obese mice. *B. pseudolongum* treatment significantly decreased the body mass, plasma triglycerides as well as visceral fat in obese mice.^36^ *B. pseudolongum* may benefit from the pancreatic exocrine insufficiency resulted in excess supply of lipids into the gut and flourish with the additional substrates. Guo et al., reported that *Bifidobacterium pseudolongum* (Bp7 and Bp8) had a protective role in colitis mice model, potentially by blocking the secretion of proinflammatory cytokines and activating the PPARγ/STAT3 pathway.^37^ However the beneficial effects attributed to *Bifidobacterium* are likely strain-specific.^38^

To our knowledge, this is the first experimental study to report that opioid treatment of CP further alters microbiome diversity, composition, and function in the already modified CP microbiome in animals. Various clinical and preclinical studies have consistently demonstrated that opioid treatments can induce dysbiosis.^39,40^ Chronic morphine administration compromises the intestinal barrier function by increasing permeability and bacterial translocation mediated by Toll-like receptors.^16,41^ We believe that the disruption in gut permeability observed is a combination of decreased localization of tight junction protein at the apical ends of the lateral membranes of intestinal epithelial cells and increased epithelial damage. In previous studies, we show that opioids treatment increase the number of epithelial apoptotic cells by inhibiting epithelial repair, implicating cellular damage in addition to disruption in tight junction localization.^42^ In line with the previous studies, the current study showed that opioids treatment alone is sufficient to induce dysbiosis with significant difference in β-diversity between control and opioids (Figure S8(a,b)), suggesting opioids treatment have an independent effect on dysbiosis without CP condition. Even though opioids have independent effect on gut microbiota, morphine or oxycodone treatment alone did not show any significant changes in pathological score (Figure S1). Opioid treatment only exacerbates pancreatitis severity when mice already developed CP through induced microbiota change. Similarly, in our germ-free acute pancreatic inflammation model, germ-free mice that received CP + MOR FMT displayed more damage than those received CP FMT (Figure 7(a–d)), indicating the dysbiotic microbiota induced by opioid treatment can lead to increased pancreatic susceptibility to injury. Further, our study showed altered β-diversity, but not α-diversity, in morphine-treated CP animals compared with CP-only animals. One plausible explanation for this observation is that the increase in α-diversity may be the result of disruption of digestive enzyme secretion due to pancreatic exocrine insufficiency and endocrine dysfunction/diabetes caused by CP.^1^ Indeed, the amylase level was reduced in CP samples. Improper digestion of carbohydrates, lipids, and other nutrients can increase the availability of nutrients for the gut microbiota, thereby increasing within-community bacterial diversity (α-diversity). Furthermore, reduced secretion of antimicrobial peptides by pancreatic acinar cells can also lead to gut microbiota overgrowth, thereby increasing α-diversity.^43^

Similarly, we also identified bacterial taxa at the species level that were associated with opioid treatment in CP animals. Moreover, virulence factors associated with opioid use and CP were also identified, which is challenging in studies using 16S amplicon sequencing. Frost et al.^3^ reported that a group of facultative pathogenic bacteria, including *Streptococcus, Escherichia.Shigell, Enterococcus*, and others, showed a 5-fold median increase in CP patient samples compared with controls. One study reported an increase in plasma endotoxin concentrations from controls to CP non-diabetic patients; however, a separate kit had to be used to detect the LPS level.^24^ Pathway analysis demonstrated that many pathways related to general amino acid metabolism and branched amino acid biosynthesis were up-regulated. This suggests that a surplus of undigested protein portions was present in the gut, driving the growth of bacteria that target that niche and resulted in greater α-diversity. A previous study discovered that short-chain fatty acid-producing bacteria were reduced in patients with CP^3;^ however, in their study, patients with CP had lower α-diversity. We speculated that this dysbiotic microbiome may be associated with an aggravated disease phenotype. To further investigate the role of the morphine-associated dysbiotic microbiome in pancreatic inflammation, we gavaged germ-free mice with fecal samples from the morphine CP group. Our results suggested that the morphine CP microbiome induced more pancreatic inflammation than the CP microbiome-treated germ-free mice in an AP setting. This result clearly emphasizes the involvement of morphine-associated dysbiosis of the microbiome in pancreatic disease. We have previously reported the role of the dysbiotic gut microbiome in morphine-induced gut barrier disruption and systemic inflammation.^16^ We also reported that the morphine microbiome is sufficient to induce gastric inflammation in the absence of morphine.^44^ However, this is the first report to suggest that the morphine-associated CP microbiome might be associated with pancreatic inflammation.

In the current study, we demonstrated that opioids increased the severity of CP and caused alterations in the gut microbiome. Oxycodone and morphine behaved similarly in terms of altering the gut microbiome, both in the control and CP conditions. Morphine- and oxycodone-treated mice were not significantly different in both α -and β-diversities, and no differentially enriched bacteria were identified in the CP groups.

The limitations of this study include a lack of inclusion of clinical samples and an unclear cause-and-effect relationship between the opioid-associated microbiome and CP severity although fecal microbial transfer recapitulated disease severity of the donor mice. Future research should be directed toward understanding the mechanisms underlying opioid- and microbiome-mediated aggravation of CP in clinical and preclinical settings. The limitation of the study also includes the cage effect in the microbiome study due to the coprophagic nature of the mouse. Future study should try to increase the number of cages to minimize the cage effect. This study also did not directly measure gut metabolites such as short chain fatty acids which will be conducted in future studies. Furthermore, harnessing microbiome manipulation to alleviate the negative effects of opioid use should be the focus of future studies.

In summary, this study reports that opioid treatment worsens the severity of CP and induces significant alterations in pain sensitivity and gut microbiome in a caerulein-induced CP mouse model.

## Materials and methods

### Experimental animals

Wild-type (WT) mice (C57BL/6J; 4–6 weeks, male) were purchased from Jackson Laboratory (Bar Harbor, Maine, USA). Animals were housed and maintained at three to five per cage and maintained on a 12-h light/dark cycle at a constant temperature (72 ± 1 °F) and 50% humidity. Food and tap water were provided *ad libitum*.

### Animal model for chronic pancreatitis and opioid administration

C57BL/6J mice were randomly divided into six groups (Control, CP, CP+ morphine, CP+ oxycodone, morphine, and oxycodone), with 10 mice per group. In each group, two cages were assigned with five animals per cage to counter the cage effect in microbiome analysis. Mice in the CP, CP+ morphine, and CP+oxycodone groups were given an intraperitoneal (i.p.) injection of sterile phosphate-buffered saline (PBS) containing caerulein (50 μg/kg body weight; Bachem, USA). Mice in the control group were administered the same volume of PBS (i.p.). Caerulein was injected once every hour for 7-hours, twice a week, over a span of 10 weeks to induce chronic pancreatitis (CP, CP+morphine, and CP+oxycodone) (Figure 1(a)). Mice in the CP+morphine group were given morphine injections from week 7 for 5 weeks at escalating doses (10, 20, 30, 40, 50 mg/kg, i.p., b.i.d), while the group was administered caerulein treatment until week 10. Mice in the CP+oxycodone group were given oxycodone injections from week 7 for 5 weeks with at escalating doses of (5, 15, 25, 35, 45 mg/kg, i.p., b.i.d.), while the group was administered caerulein treatment until week 10. The morphine and oxycodone groups received either morphine or oxycodone treatment from week 7 for 5 weeks, respectively. At week 6, 4 mice from the control group and 6 mice from the CP group were euthanized to evaluate the progression of chronic pancreatitis. The remaining mice were euthanized at Week 11. Mice kept in the same group were randomized (group-wise) to discount the cage effect in microbiome studies. Animals were euthanized at different time points according to protocols approved by the University of Miami Animal Care Committee. The mice were euthanized using CO_2_ chambers and death was ensured by cervical dislocation. Gut content was collected aseptically. Pancreatic tissues were formalin-fixed for paraffin embedding and histochemical analysis. The severity of CP and regeneration were compared between the groups.

### Histology and Sirius red staining and measurements

The mouse pancreases were fixed in 10% formalin and embedded in paraffin. The sections (5 μm) were cut using a microtome, stained with hematoxylin and eosin, and assessed using a microscope (Leica Microsystems, Germany) at an original magnification of 10 × 10 and processed in Adobe Photoshop. Five microscopic pancreatic fields covering all tissue sections were quantified by a blinded morphologist using a scoring system, as described previously^45^ with modifications. Areas of abnormal pancreatic tissue architecture were the first parameter and graded as follows: 0 absent to 10 major of the total parenchyma affected. Within these areas, glandular atrophy, presence of pseudo-tubular complexes, and fibrosis were graded from 0: absent to 10: maximum severity, and the total score was compared between the groups. Tissue sections were stained using Sirius Red staining solution (Chondrex Inc., WA, USA), according to the manufacturer’s instructions. The Sirius red-stained area was quantified using ImageJ software by selecting stained fibers in five fields at a magnification of 10 × 10 under a light microscope.

### Western blotting

To analyze protein expression, parts of mice pancreases were lysed using RIPA lysis buffer (Boston Bioproducts, MA, USA) containing protease and phosphatase inhibitors (Roche, Switzerland), and protein concentration was estimated using the BCA protein estimation assay (Thermo Scientific, MA, USA). Equal amounts of protein were separated by SDS-PAGE and transferred to the nitrocellulose membrane. Blots were probed with antibodies against pancreatic amylase (ab199132), alpha SMA (ab5694) and beta tubulin (ab179513) (Abcam, UK, 1:1000 dilution), after washing and re-probed with the respective secondary antibody (Abcam, UK, 1:10000 dilution). The bands were visualized using super signal West Dura Extended Duration Subject (34075- Thermo Fisher) in a Chemi-Doc (Bio-Rad).

### Immunofluorescence

Distal small intestinal section was harvested and fixed in 10% formalin folllowed by embedding in paraffin wax. For immunostaining with tight junction protein, ~8 μm tissue section were deparaffinized and rehydrated by passing through a series of alcohol gradient. Heat antigen retrieval was performed using citrate antigen retrieval buffer (pH 6.0). After blocking the tissue section with 5% BSA, staining was done with Claudin-1 antibody (1:100) overnight at 4°C. Tissue sections were incubated with Alexa fluor 488-conjugated secondary antibody (1:800) for 1 h at room temperature and ProLong Gold antifade reagent with DAPI was used for mounting coverslip on tissue section. Imaging was done with Leica fluorescence microscope (Leica Microsystems, Germany).

### Pain measurement using tail flick and Randall-Selitto test

Morphine hyperalgesia in mice with CP was measured by the latency of withdrawal using tail-flick assays.^46^ Briefly, CP mice were intraperitoneally injected with morphine or oxycodone twice daily for 5 weeks at 12-hour intervals. Pain assessment was performed before morphine or oxycodone administration in the morning during the 12-hour light cycle. The phosphate-buffered saline group was used as the control group. Withdrawal latencies of the tail from a radiant heat source were measured using a tail flick. The voltage of the light source was adjusted to achieve a baseline latency between 2 and 3 seconds. The cutoff time was 10 seconds to avoid tissue damage. The nociceptive withdrawal threshold was assessed using a Randall-Selitto electronic algesimeter (IITC 2500 Digital Paw Pressure Meter, IITC Life Science, Woodland Hills, CA, USA). Before the test, each animal received 5 minutes of handling to adjust to manipulation, and was then placed on a soft cotton cloth and carefully immobilized with the same hand used to hold the tested paw. The test consisted of the application of an increasing mechanical force, in which the tip of the device was applied to the medial portion of the plantar or dorsal surfaces of both the fore and hind paws until a withdrawal response occurred. The point of application was marked with ink to maintain its location over repeated trials. To prevent skin damage, the maximum force applied was limited to 250 g.

### Fecal microbiota transplantation into germ free mice and myeloperoxidase (MPO) assay

To investigate whether morphine-induced gut microbiota could affect pancreatic necrosis and inflammation, we first established a fecal microbiota transplantation (FMT) model of actuate pancreatitis in a germ-free mouse. Fecal samples from control, CP and CP + Morphine mice were collected and suspended in 1 ml sterile PBS, filtered through a 70 μm cell strainer, and centrifuged at 6000 g for 20 min.^44^ The supernatant was gavaged to germ-free mice 2 times on day 1 and 3, with four to five mice per group. On day 4, all groups of mice were administered an intraperitoneal injection of a suboptimal dose of caerulein (5 μg/kg body weight at 7 hourly intervals). Wild-type mice were simultaneously challenged with a suboptimal dose of caerulein to compare pancreatic injury with germ-free mice. All mice were euthanized 12 hours after caerulein injection. Pancreases and lungs were collected and parts of the pancreas were formalin-fixed for paraffin embedding and histochemical analysis. Five microscopic pancreatic fields, covering all tissue sections, were quantified in a blinded manner. Areas of pancreatic tissue damage were graded as follows: 0: absent to 10 major, and the total score was compared between groups. The MPO assay (Abcam, UK) was performed on the pancreas and lung extracts of groups of mice. Briefly, pancreases and lungs were rapidly homogenized in four volumes of MPO assay buffer. Centrifugation was performed at 4°C to remove insoluble materials and subjected to the MPO assay according to the manufacturer’s standard protocol (MAK068, Sigma-Aldrich). MPO activity was expressed as milliunits/mg.

### DNA extraction and quantification

DNA was isolated from fecal samples using a DNeasy 96 PowerSoil Pro QIAcube HT Kit with a QIAcube HT liquid-handling machine (Qiagen, Maryland, USA). Extracted DNA samples were quantified with the GloMax Plate Reader System (Promega) using QuantiFluor® dsDNA System (Promega) chemistry.

### Library preparation and sequencing methods

Library preparation and sequencing were performed by CosmosID Inc. DNA libraries were prepared using the Nextera XT DNA Library Preparation Kit (Illumina) and IDT Unique Dual Indexes, with a total DNA input of 1ng. Genomic DNA was fragmented using a proportional amount of Illumina Nextera XT fragmentation enzyme. Unique dual indices were added to each sample, followed by 12 cycles of PCR to construct the libraries. DNA libraries were purified using AMpure magnetic beads (Beckman Coulter) and eluted using the QIAGEN EB buffer. DNA libraries were quantified using a Qubit 4 fluorometer and a Qubit dsDNA HS Assay Kit. Libraries were sequenced on an Illumina HiSeq X platform at 2 × 150 bp.

### Bioinformatics analysis

The system utilizes a high-performance data-mining k-mer algorithm that rapidly disambiguates millions of short-sequence reads into discrete genomes engendering particular sequences. The pipeline had two separable comparators: the first consisted of a pre-computation phase for reference databases, and the second was a per-sample computation. The inputs to the pre-computation phase were databases of reference genomes, virulence markers, and antimicrobial resistance markers, which were continuously curated by CosmosID. The output of the pre-computational phase is a phylogenetic tree of microbes, together with sets of variable-length k-mer fingerprints (biomarkers) uniquely associated with distinct branches and leaves of the tree. The second per-sample computational phase searched hundreds of millions of short-sequence reads against fingerprint sets. This query enabled the sensitive yet highly precise detection and taxonomic classification of microbial NGS reads. The resulting statistics were analyzed to return fine-grain taxonomic and relative abundance estimates for the microbial NGS datasets. To exclude false-positive identifications, the results were filtered using a filtering threshold derived from internal statistical scores that were determined by analyzing a large number of diverse metagenomes. The same approach was applied to enable sensitive and accurate detection of genetic markers for virulence and antibiotic resistance. The taxonomic identification results are summarized in Table S1.

For functional classification, initial QC, adapter trimming, and preprocessing of metagenomic sequencing reads were performed using BBduk.^47^ The quality-controlled reads were then subjected to a translated search against a comprehensive and nonredundant protein sequence database, UniRef 90. The UniRef90 database, provided by UniProt,^48^ represents a clustering of all non-redundant protein sequences in UniProt such that each sequence in a cluster aligns with 90% identity and 80% coverage of the longest sequence in the cluster. The mapping of metagenomic reads to gene sequences was weighted by mapping quality, coverage, and gene sequence length to estimate community-wide weighted gene family abundances, as described by Franzosa et al..^49^ Gene families were annotated to MetaCyc^50^ reactions (Metabolic Enzymes) to reconstruct and quantify MetaCyc metabolic pathways in the community, as described by Franzosa et al..^49^ Gene families were also regrouped into Kyoto Encyclopedia of Genes Orthogroups^51^ to get an overview of KEGG pathways in the community. Furthermore, the UniRef_90 gene families were also regrouped to GO terms^52^ to obtain an overview of GO functions in the community. Lastly, to facilitate comparisons across multiple samples with different sequencing depths, the abundance values were normalized using Total-sum scaling (TSS) normalization to produce “Copies per million” (analogous to TPMs in RNA-Seq) units. MicrobiomeAnalyst^53^ was used to generate alpha diversity, heatmap, and LefSe plots. The threshold of the logarithmic LDA score for discriminative features was set to 2. The cutoff for the false discovery rate-adjusted p-value (q-value) was set to 0.1 for LefSe analysis.

### Statistical analysis

The Mann-Whitney test or Kruskal-Wallis test was used to detect whether α diversity differed across treatments. Permutational multivariate analysis of variance (PERMANOVA) was used to determine whether β-diversity differed across treatments. The Benjamini-Hochberg method was used to control the false discovery rate (q-value). All other statistical analyses were performed using GraphPad Prism software. Data are expressed as mean ± standard error of the mean (SEM) and compared using the unpaired Student’s t-test and one-way analysis of variance (ANOVA). T-tests were used to determine differences between CP and control mice, and ANOVAs were used to assess differences between three or more groups. Statistical significance was set at *p* < .05.

### Study approval

All the animal experimental protocols were approved by the Institutional Animal Care and Use Committee of the University of Miami. All procedures were conducted in accordance with the guidelines set forth by the National Institutes of Health Guide for the Care and Use of Laboratory Animals. Animal studies were reported in compliance with the ARRIVE guidelines.^54^

## Supplementary Material

Supplemental Material

Supplemental table_S1toS6.xlsx

## Data Availability

Sequence data were deposited in the Biostudies database (https://www.ebi.ac.uk/biostudies/) under accession number S-BSST1080 (https://www.ebi.ac.uk/biostudies/studies/S-BSST1080).
