# Supplementary figures and images for: Prescription opioids induced microbial dysbiosis worsens severity of chronic pancreatitis and drives pain hypersensitivity
Source: Gut Microbes. 2024 Feb 8;16(1):2310291. doi: 10.1080/19490976.2024.2310291 (PMC10857465; doi:10.1080/19490976.2024.2310291)

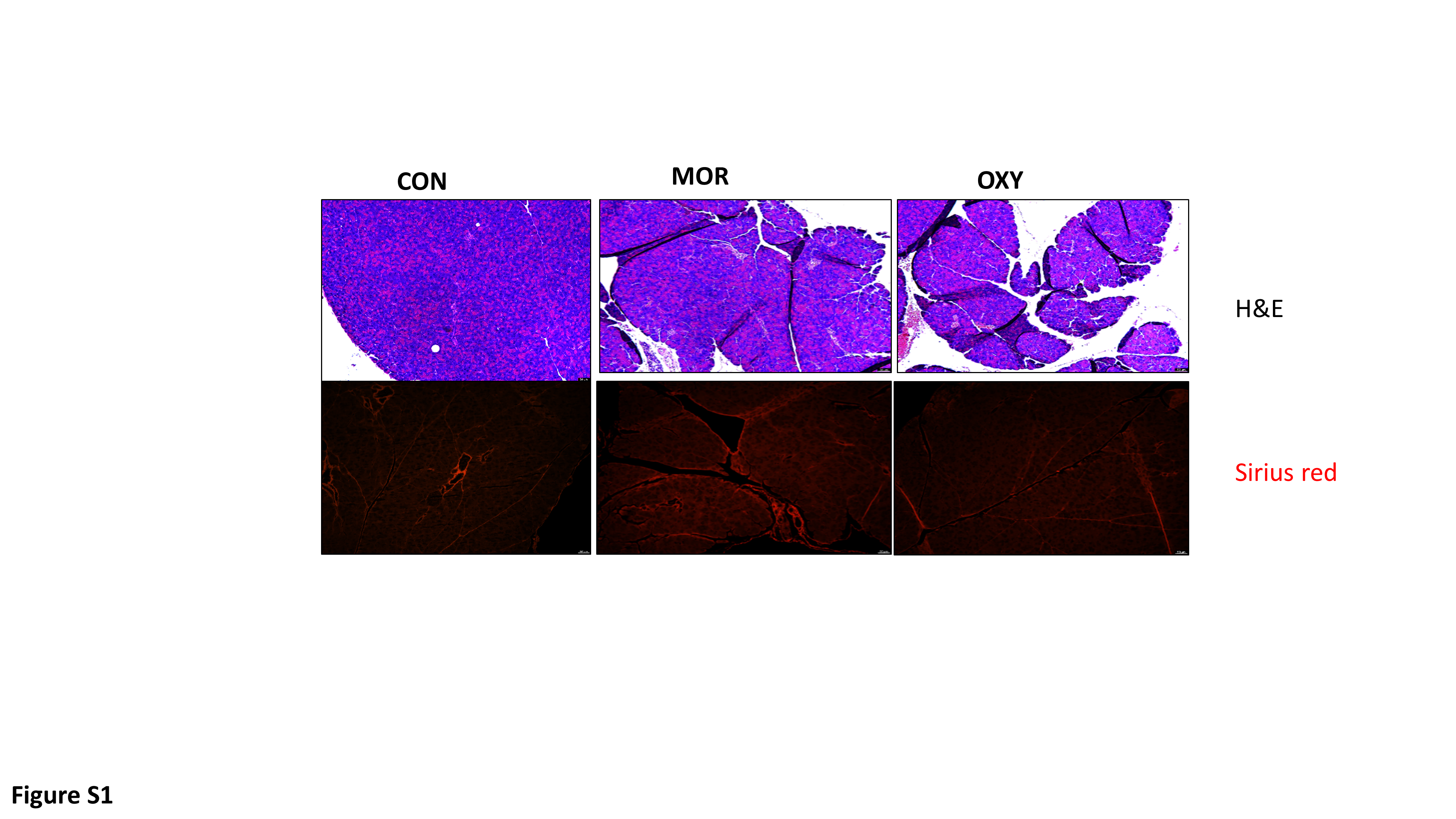

Supplement: Supplemental Material [file KGMI_A_2310291_SM7907.zip › Fig S1.TIF]

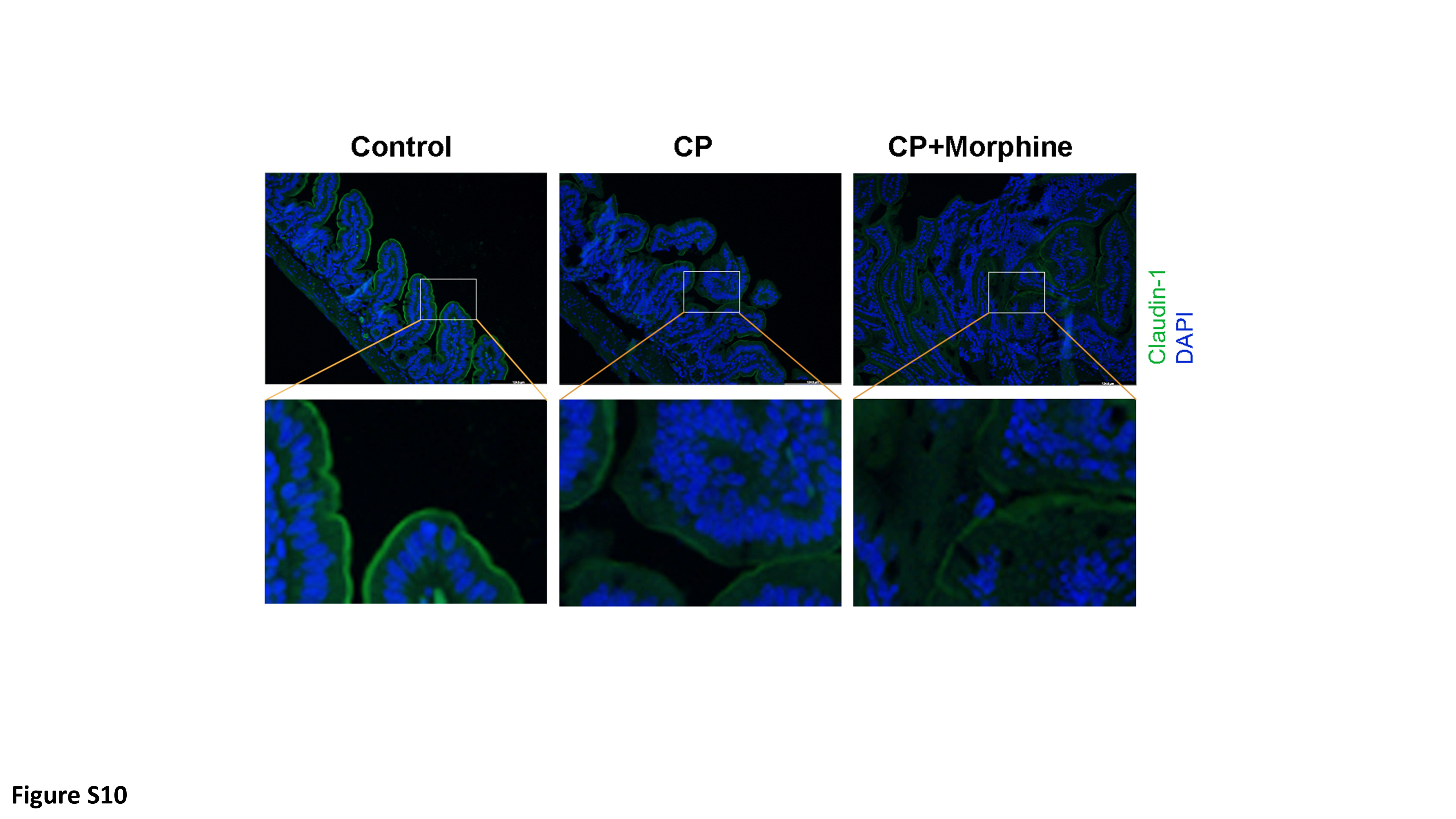

Supplement: Supplemental Material [file KGMI_A_2310291_SM7907.zip › Fig S10.TIF]

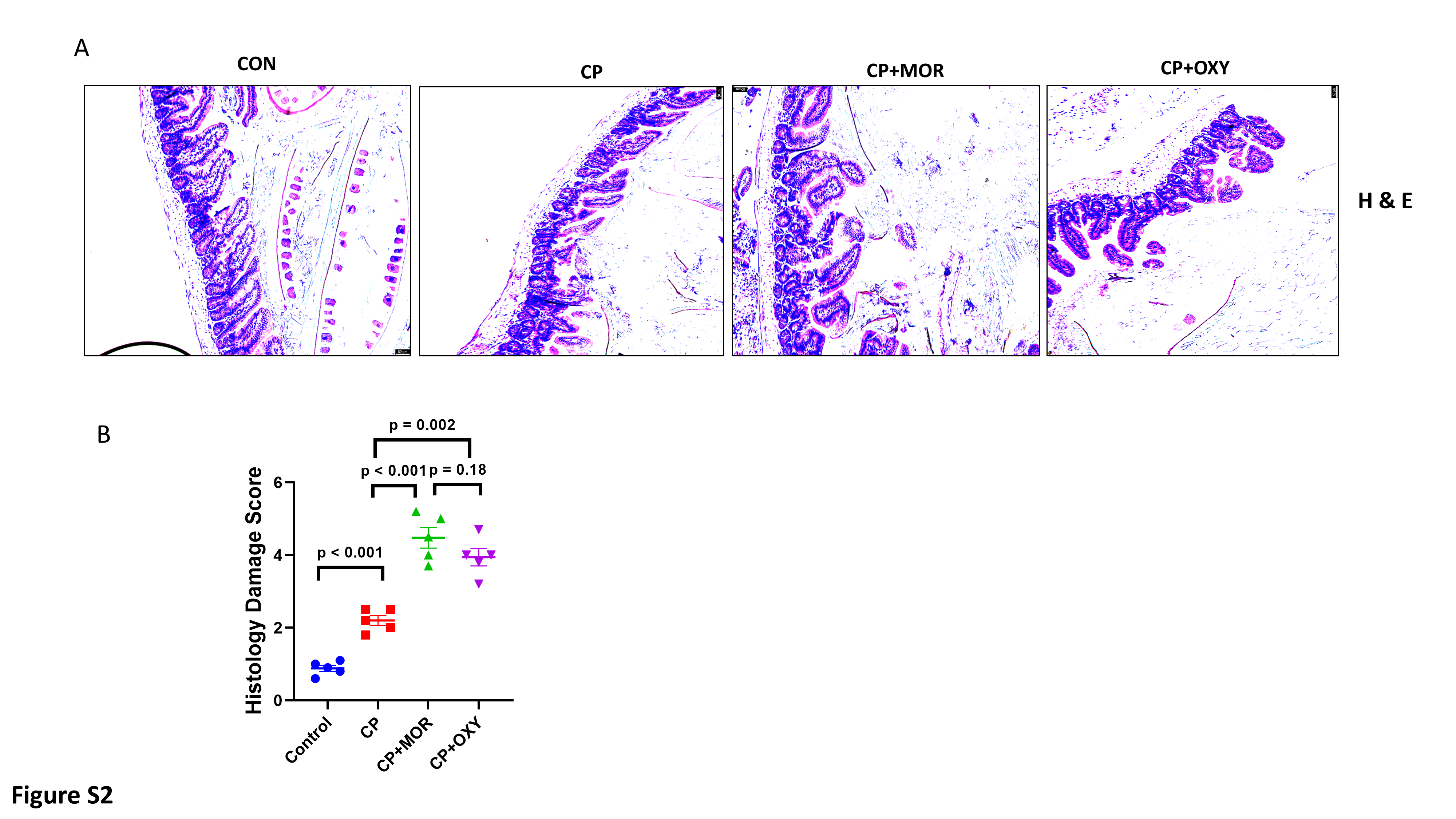

Supplement: Supplemental Material [file KGMI_A_2310291_SM7907.zip › Fig S2.TIF]

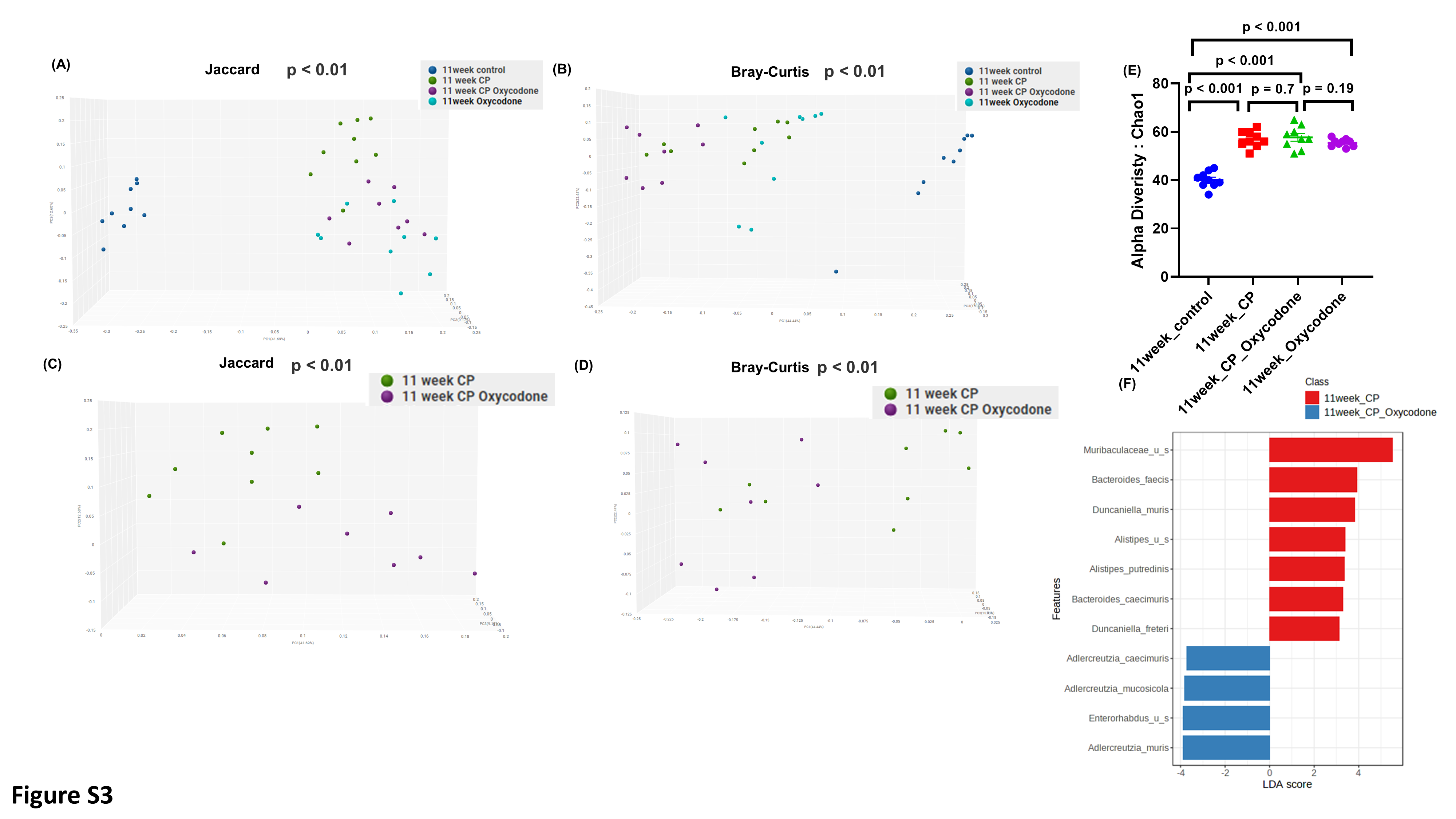

Supplement: Supplemental Material [file KGMI_A_2310291_SM7907.zip › Fig S3.TIF]

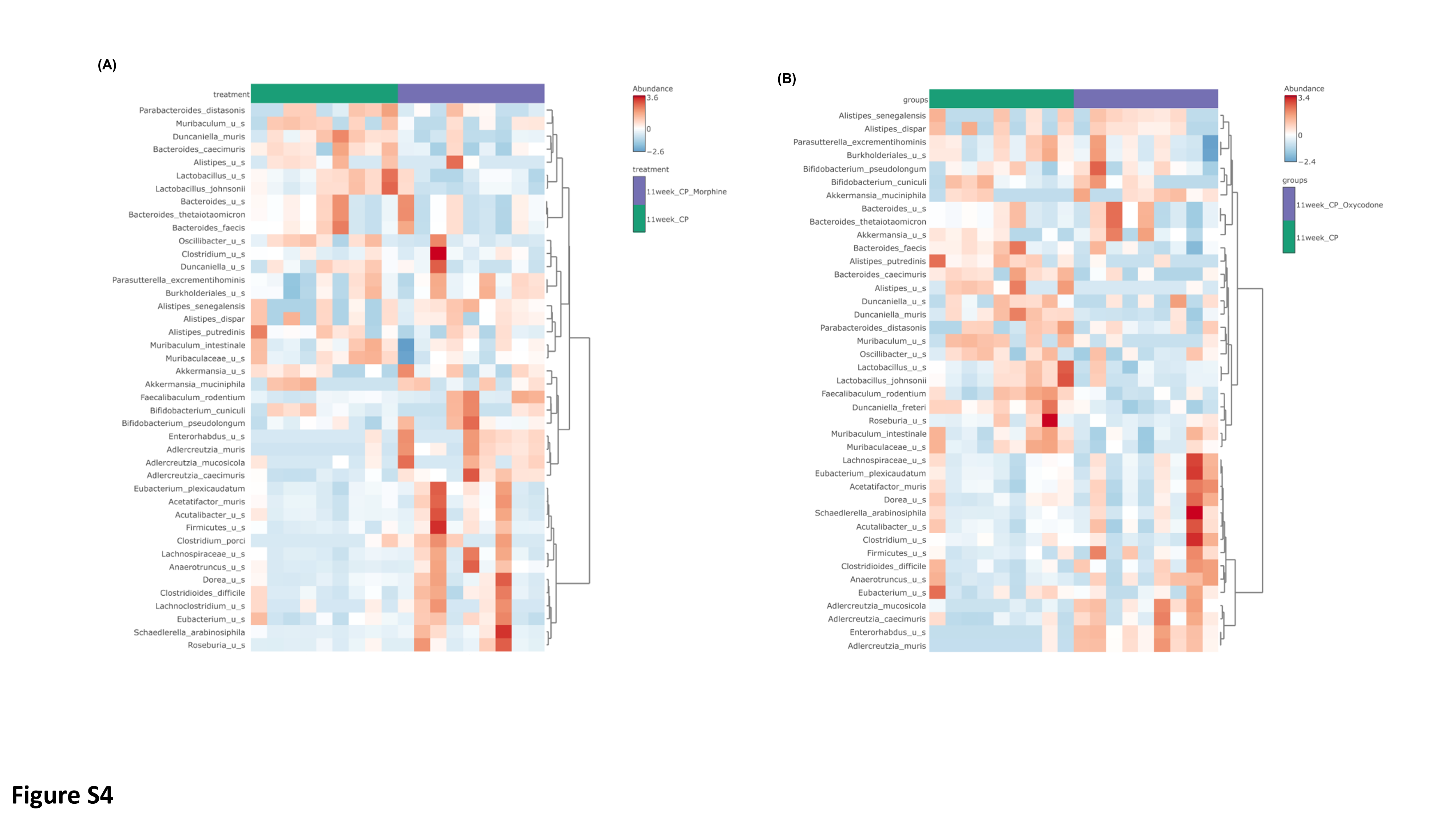

Supplement: Supplemental Material [file KGMI_A_2310291_SM7907.zip › Fig S4.TIF]

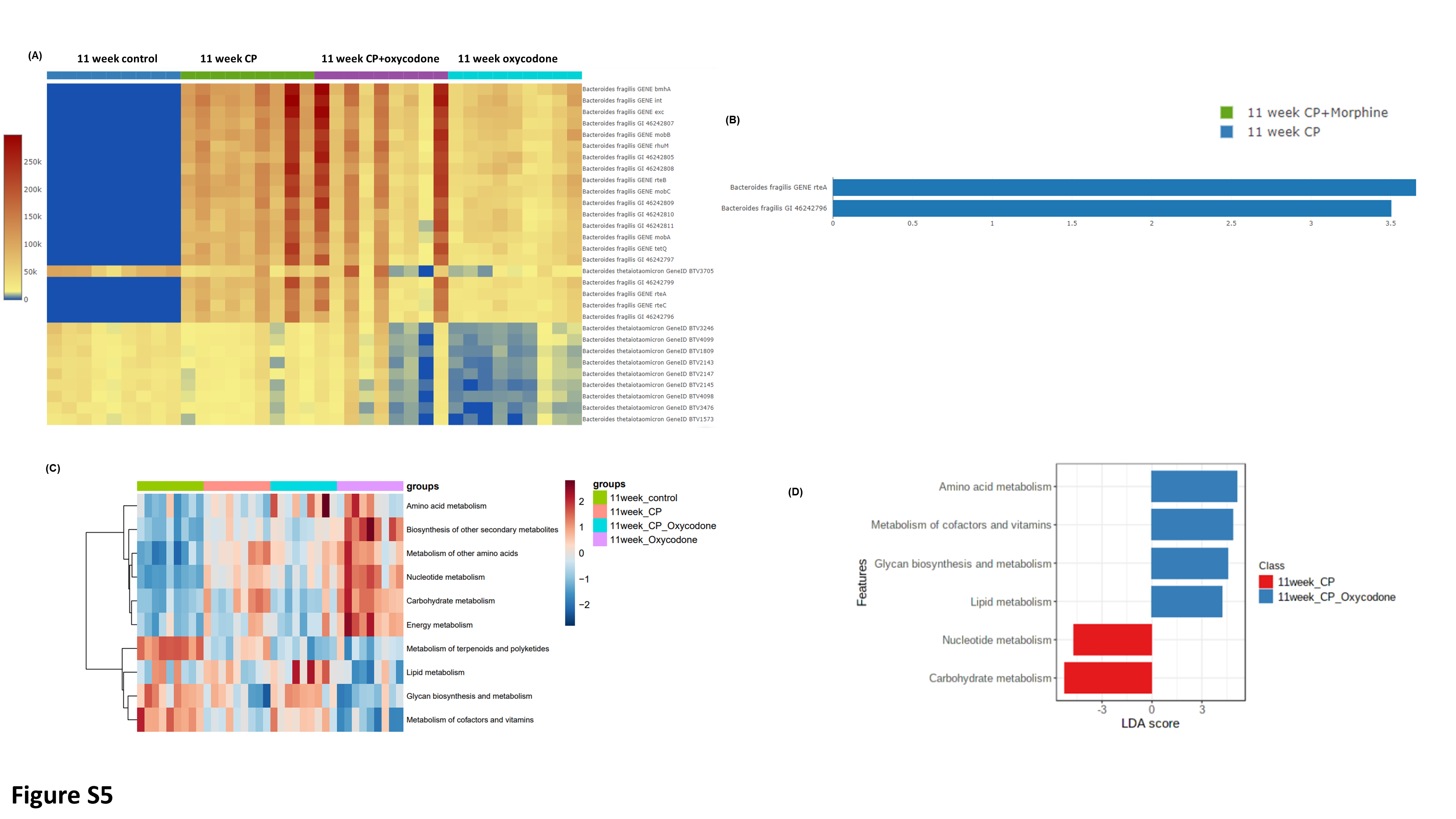

Supplement: Supplemental Material [file KGMI_A_2310291_SM7907.zip › Fig S5.tif]

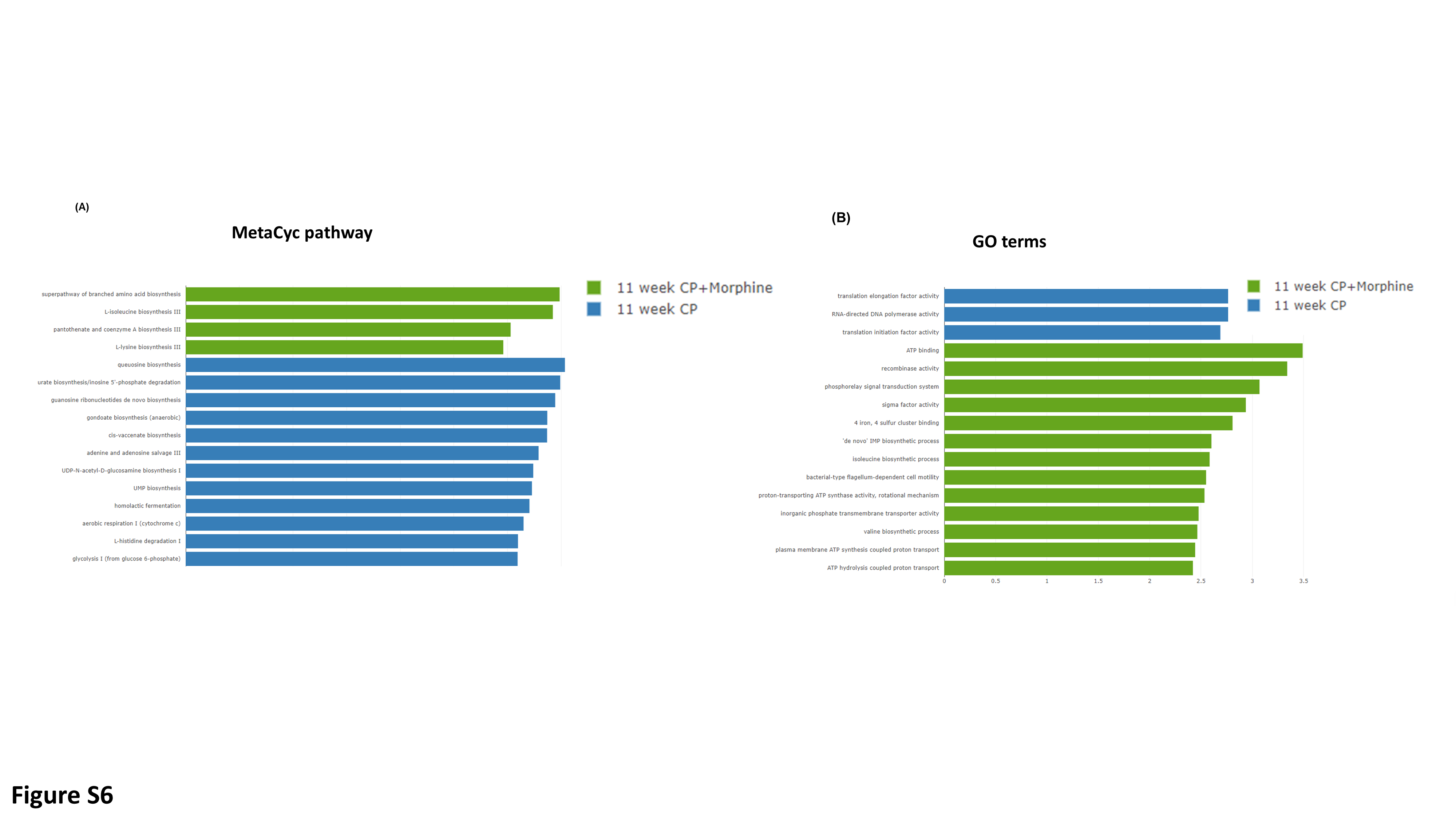

Supplement: Supplemental Material [file KGMI_A_2310291_SM7907.zip › Fig S6.TIF]

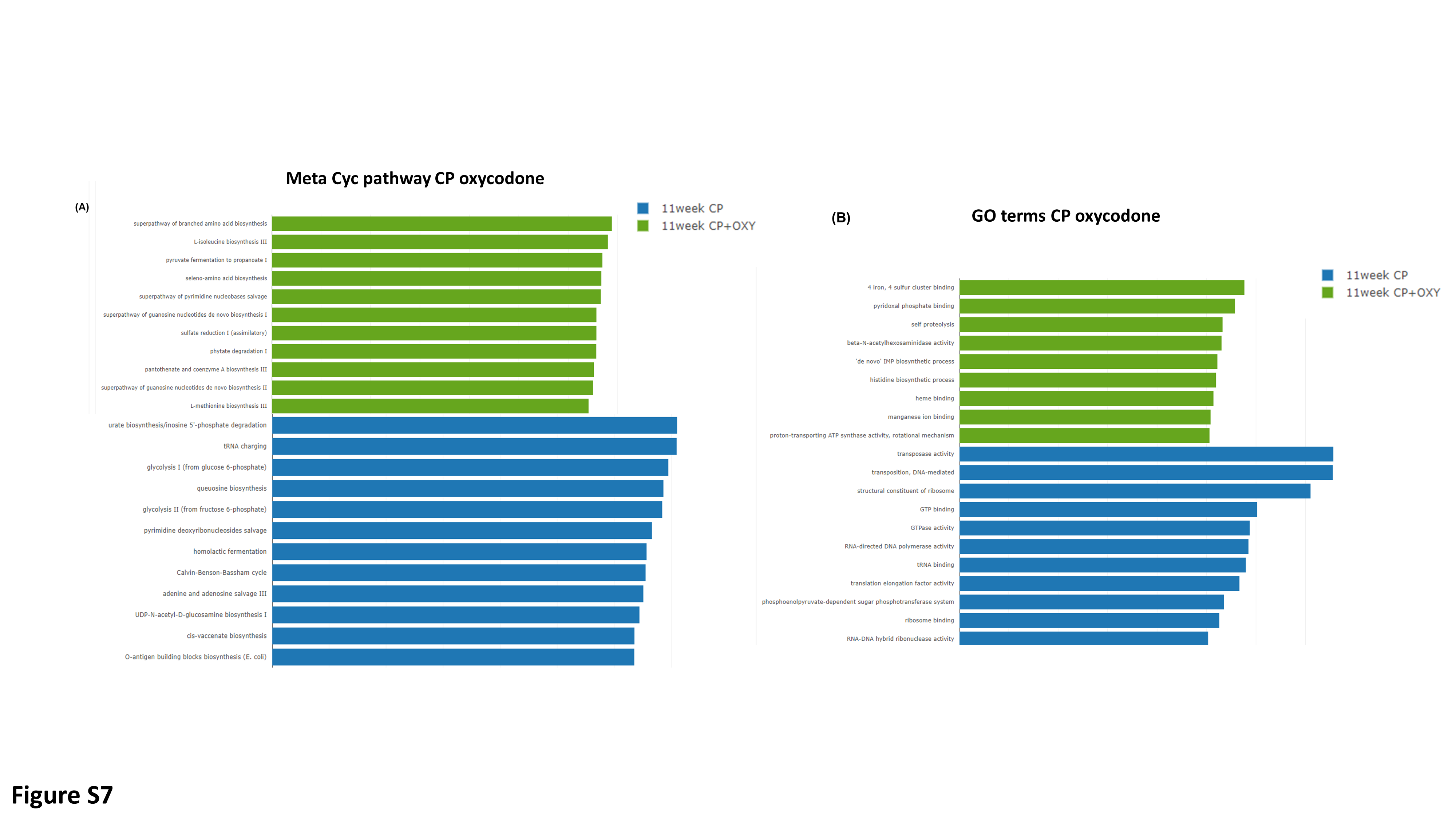

Supplement: Supplemental Material [file KGMI_A_2310291_SM7907.zip › Fig S7.TIF]

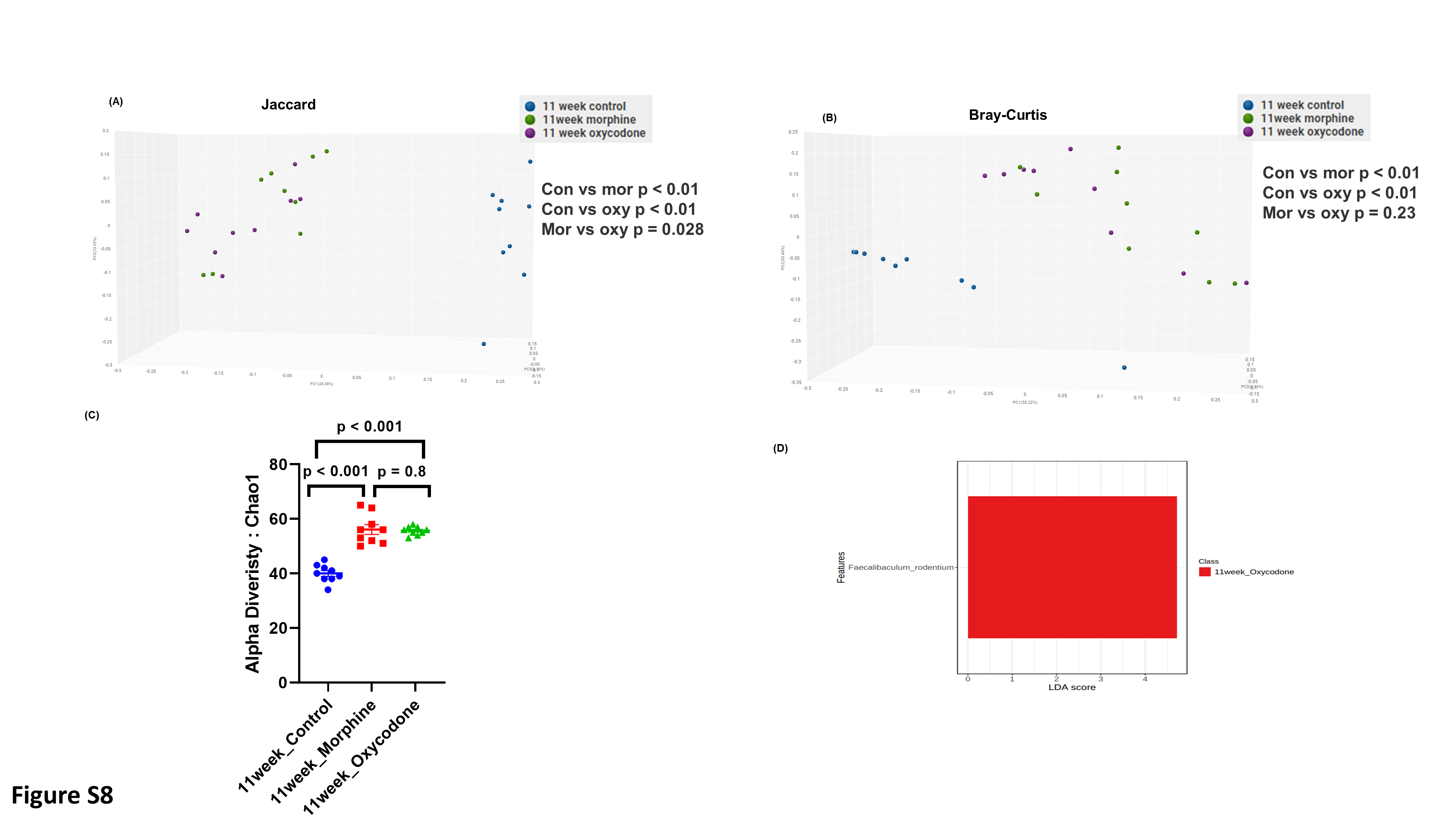

Supplement: Supplemental Material [file KGMI_A_2310291_SM7907.zip › Fig S8.TIF]

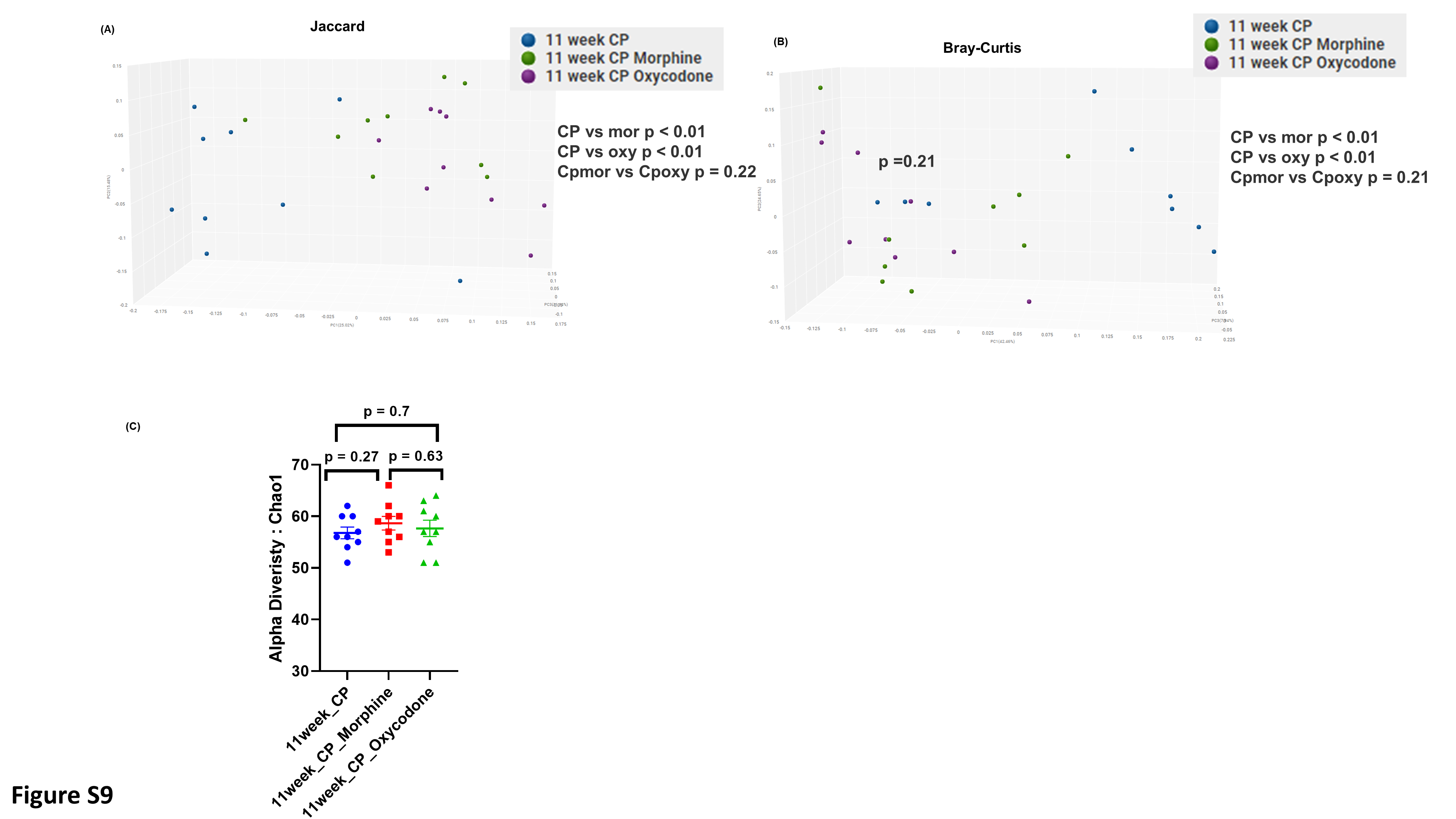

Supplement: Supplemental Material [file KGMI_A_2310291_SM7907.zip › Fig S9.TIF]
